# Underwater and traction-assisted endoscopic submucosal dissection for laterally spreading tumors in colonic postoperative blind loops

**DOI:** 10.1055/a-2885-8195

**Published:** 2026-06-23

**Authors:** Mengyuan Zhang, Xin Wei, Qing Shao, Zhaohui Liu, Jing Wang, Tao Xie, Hui Wang

**Affiliations:** 1 Department of Gastroenterology, Rizhao 549615 People's Hospital, Rizhao, Shandong, China; 2 Department of General Surgery 549615 Rizhao People's Hospital, Rizhao, Shandong, China

A 74-year-old woman with a history of left hemicolectomy for transverse colon cancer 3 years before underwent colonoscopy. Examination revealed a blind loop at the side-to-end anastomosis, 40 cm from the anal verge, within which a non-granular laterally spreading tumor (LST) measuring approximately 4×3 cm was noted. Blue laser imaging revealed Type IV and Type III pit patterns ( Fig. 1 ).

**Fig. 1:**
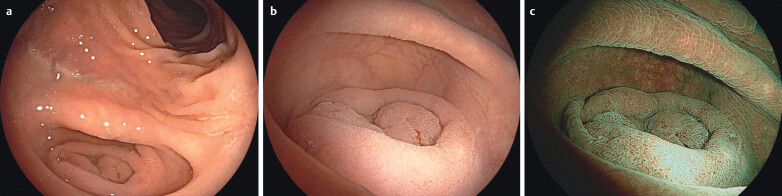
An LST measuring approximately 4×3 cm within the blind loop.

The procedure was performed under deep sedation with propofol. Circumferential treatment of the entire blind loop was carried out as follows: A transparent cap was attached to the tip of a 290 T endoscope. Using a dual knife (Olympus Medical, Tokyo, Japan), a circumferential incision was made around the lesion. A submucosal injection of sodium hyaluronate mixed with methylene blue and normal saline was administered, resulting in a good lift of the lesion. The edges of the lesion were incised, and the lesion was resected using the underwater method ( Fig. 2a ). Significant adhesion was noted at the base of the lesion. An internal traction clip was applied to grasp both ends of the lesion, providing retraction toward the oral side to facilitate complete resection ( Fig. 2b ). The resection revealed that the blind loop involved the serosal layer ( Fig. 3 ). After full-thickness resection, the defect was closed using the origami method with metallic clips. The operative duration was 2 hours. The key steps of the procedure are demonstrated in the attached video ( Video 1 ).

**Fig. 2:**
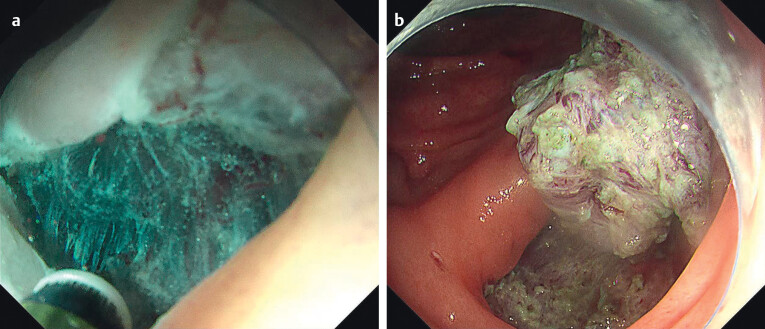
( **a** ) Underwater ESD. ( **b** ) Lesion approximation and traction.

**Fig. 3:**
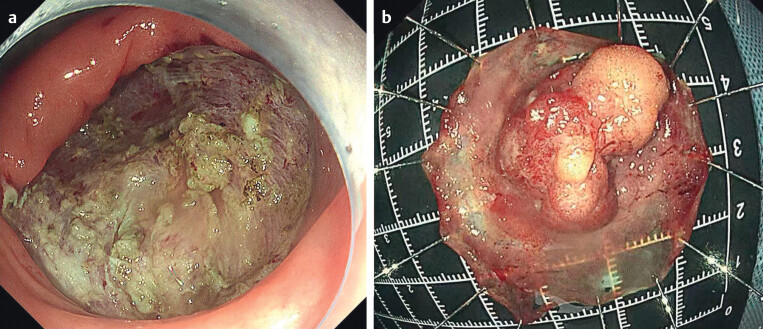
An endoscopic view demonstrating the postoperative mucosal defect and the corresponding en bloc resected specimen.

**Video 1** Endoscopic removal of an LST within a postoperative colonic blind loop.


The patient was kept fasting for 48 hours postoperatively and received antibiotic therapy. There were no postoperative complaints such as abdominal pain or hematochezia, and the patient was discharged on postoperative day 3. Pathological diagnosis: Tubular adenoma with focal high-grade intraepithelial neoplasia (intramucosal carcinoma). Both the circumferential and deep margins of the specimen are free of lesions.

Endoscopic management of lesions in postoperative colonic blind loops is considered challenging due to excessive mobility and poor endoscopic visualization. The combined underwater and traction-assisted endoscopic submucosal dissection technique enabled complete endoscopic resection, obviating secondary surgery and reducing patient morbidity.

Endoscopy_UCTN_Code_TTT_1AO_2AG_3AD

## Informed consent

Written informed consent was obtained from the patient to publish these images and the video.

